# Frequency Response of Graphene Electrolyte-Gated Field-Effect Transistors

**DOI:** 10.3390/s18020494

**Published:** 2018-02-07

**Authors:** Charles Mackin, Elaine McVay, Tomás Palacios

**Affiliations:** Massachusetts Institute of Technology, Department of Electrical Engineering and Computer Science, 77 Massachusetts Avenue, Cambridge, MA 02139, USA; cmackin@mit.edu (C.M.); emcvay@mit.edu (E.M.)

**Keywords:** ambipolar transistor, chemical and biological sensors, device modeling, frequency response, small-signal model, electrophysiology, graphene field-effect transistor (GFET), graphene electrolyte-gated field-effect transistor (EGFET)

## Abstract

This work develops the first frequency-dependent small-signal model for graphene electrolyte-gated field-effect transistors (EGFETs). Graphene EGFETs are microfabricated to measure intrinsic voltage gain, frequency response, and to develop a frequency-dependent small-signal model. The transfer function of the graphene EGFET small-signal model is found to contain a unique pole due to a resistive element, which stems from electrolyte gating. Intrinsic voltage gain, cutoff frequency, and transition frequency for the microfabricated graphene EGFETs are approximately 3.1 V/V, 1.9 kHz, and 6.9 kHz, respectively. This work marks a critical step in the development of high-speed chemical and biological sensors using graphene EGFETs.

## 1. Introduction

Graphene consists of an atomically thin layer of sp^2^-bonded carbon atom arranged in a hexagonal lattice [1,2,3,4]. Although graphene lacks a bandgap and is ill suited for traditional digital logic circuits, graphene exhibits a number of promising electrical, optical, mechanical, and chemical properties making it well suited for a variety of chemical and biological sensing applications. Key properties include high carrier mobilities [5], low optical absorption [6,7], mechanical strength and flexibility [6,7,8,9,10], as well as chemical stability [11,12,13,14]. Because graphene exhibits excellent chemical stability and does not form a native oxide like its silicon metal-oxide-semiconductor field-effect transistor (Si-MOSFET) counterparts, graphene electrolyte-gated field-effect transistors (EGFETs) can take full advantage of the ultrahigh gate capacitance resulting from the electric double layer phenomenon [15]. Excellent chemical stability also enables a direct interface with many chemical and biological environments [11,13,14,16]. This ultra-high gate capacitance, which is in the order of μF/cm^2^, provides graphene EGFETs with excellent transconductance performance [17,18]. It also raises the concern of impaired frequency response due to high parasitic gate-source and gate-drain capacitances. This is especially concerning in the case of graphene EGFETs because transconductance performance is greatly enhanced by recessing device passivation such that portions of the source and drain contacts are exposed [19].

A number of models have been developed describing the DC behavior of graphene FETs, including two models which accurately describe DC characteristics of graphene EGFETs [20,21,22,23,24,25]. Little work, however, has been reported regarding the AC capabilities of graphene EGFETs. Accurate frequency response models are critical in the development of graphene EGFETs for applications such as electrophysiology sensors. Previous studies have shown graphene EGFETs capable of providing low-noise signal transduction for neuronal action potentials [16,26]. These studies, however, employ graphene EGFETs merely for source-drain current modulation (i.e., as tunable resistors) and therefore result in greatly attenuated action potential signals.

The aim of this paper is two-fold: to advance graphene EGFET sensing technology for applications such as electrophysiology by demonstrating the ability of graphene EGFETs to operate as functional amplifiers, and to develop the corresponding small-signal frequency-dependent model necessary to understand the amplification characteristics. Development of high-speed graphene EGFETs may find use as chemical sensors in applications such as high-throughput microfluidics [27]. Characterization of frequency response for the first time also enables entirely new sensing technologies, such as electronic tongues, where changes in the frequency response (i.e., spectral content) may be analyzed to sense changes in solution composition. This is closely related to spectroscopic and time constant techniques employed in electronic nose technologies [28,29,30]. Lastly, frequency response characterization is critical in avoiding signal aliasing when interfacing graphene EGFET sensors with analog-to-digital (ADC) converters in the development of practical sensor readout systems [31].

## 2. Graphene EGFET Background

### 2.1. Graphene-Electrolyte Interface Capacitance

Immersion of an electrode in an electrolyte results in the accumulation of ions at the electrode/electrolyte interface due to differences in electrochemical potential. This phenomenon is termed the electric double layer. The amount of charge stored in the electric double layer may be controlled—much like a capacitor—by changing the electric potential of the electrode with respect to the electrolyte potential [32]. In the case of graphene, the capacitance of the electric double layer is large enough that accurately modeling the graphene-electrolyte interface capacitance requires inclusion of the graphene quantum capacitance [15]. Quantum capacitance is proportional to the density of states and places an upper bound on the capacitance achievable in two-dimensional materials such as graphene. The graphene quantum capacitance is critical to understanding the effects of parasitic capacitances on graphene EGFET frequency response. Equations (1) and (2) describe the graphene quantum capacitance [18].

(1) $$C_{Q}=\frac{2q^{2}}{\hbar v_{F}\sqrt{\pi }}{\left(\left|n_{G}\right|+\left|n^{*}\right|\right)}^{1/2}$$

(2) $$n_{G}={\left(\frac{qV_{ch}}{\hbar v_{F}\sqrt{\pi }}\right)}^{2}$$

Here ℏ is the reduce Planck constant, $v_{F}$ is the Fermi velocity, $n_{G}$ is the carrier concentration induced by the gate voltage, $n^{*}$ is the effective charged impurity concentration, and $V_{ch}$ is the electric potential of the graphene channel.

**Figure 1:**
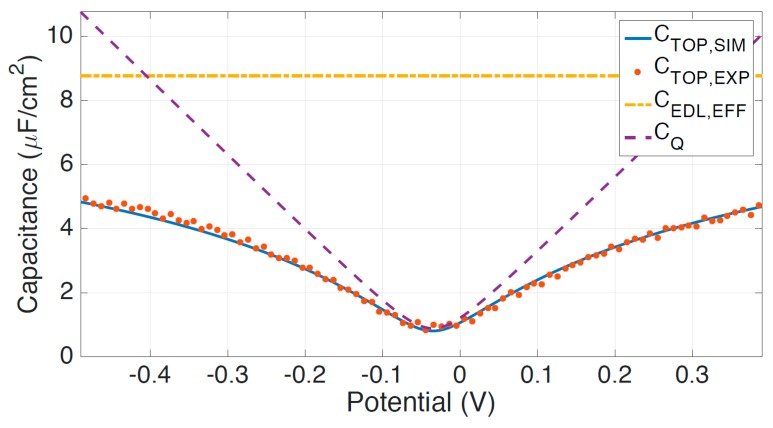
Measured top-gate capacitance as a function of potential using electrochemical impedance spectroscopy. C_TOP,EXP_ (**dotted red**) is the measured top-gate capacitance, C_EDL,EFF_ (**dash-dot yellow**) is the effective electric double layer capacitance, C_Q_ (**dashed purple**) is the quantum capacitance, and C_TOP,SIM_ (**blue**) is the simulated top-gate capacitance. C_TOP,SIM_ consists of the effective double layer capacitance in series with the graphene quantum capacitance.

Graphene-electrolyte interface capacitances have been experimentally examined in numerous works [17,19,33,34]. The graphene/electrolyte interface capacitance may be modeled using a parallel plate capacitor in series with the graphene quantum capacitance. As a hydrophobic material, graphene repels aqueous electrolytes resulting in an angstrom-scale gap between the electrolyte and graphene surface. This forms a parallel plate capacitor, which reduces the complex voltage-dependence capacitance typical of electric double layers. Ultimately, graphene-electrolyte interface capacitances typically range from approximately 1–5 μF/cm^2^ as measured by electrochemical impedance spectroscopy (EIS), and shown in Figure 1. The mean value of the graphene-electrolyte interface capacitance is 3 μF/cm^2^, which is useful for approximations.

### 2.2. Graphene EGFET DC Current-Voltage Model

**Figure 2:**
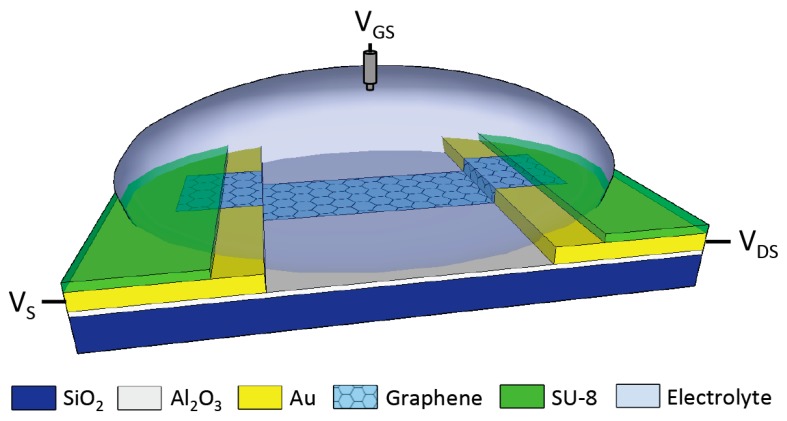
Graphene electrolyte-gated field-effect transistors (EGFET) with recessed top-gate capacitance due to non-self-aligned source/drain passivation. The gate voltage is applied using a reference electrode.

Graphene EGFETs are comprised of two conductive contacts, typically metals, connected by a graphene channel region. The bulk of the metal leads are passivated to reduce leakage current through the electrolyte due to applied gate voltage. The graphene channel region is gated directly through the electrolyte using a reference or pseudo-reference electrode. Figure 2 depicts the typical layout for a graphene EGFET and experimental setup.

The current at any given position in the graphene channel is determined by the product of carrier concentration and the carrier drift velocity, which is scaled appropriately by the elementary charge and channel width. This principle, in conjunction with current continuity, enables calculation of the graphene EGFET current and the corresponding channel potential profile. The channel current is given by the following equation [19,20,35]:

(3) $$I_{DS}=\frac{q\mu \frac{W}{L}\int_{I_{DS}R_{C}}^{V_{DS}-I_{DS}R_{C}}\sqrt{n_{o}^{2}+{\left[C_{TOP}\left(V\right)\left[V_{GS,TOP}-V-V_{o}\right]/q\right]}^{2}}dV}{1+\left|\frac{\mu \left(V_{DS}-2I_{DS}R_{C}\right)}{Lv_{sat}}\right|}$$

where W is the channel width and L is the channel length, μ is the carrier mobility, and *V* is the channel potential which is a function of position, $n_{o}$ is the minimum carrier concentration [36,37], $C_{TOP}$ is the top-gate capacitance, $V_{GS,TOP}$ is the applied top gate voltage, $v_{sat}$ is the saturation velocity, $R_{C}$ is the contact resistance, and *V_o_* represents the gate voltage at the Dirac point. This particular model assumes carrier mobilities are equal for holes and electrons and independent of the carrier concentration. Contact resistances are assumed to be symmetric. It is also important to note that chemical and biological sensors employing graphene EGFETs are typically biased at low voltages to avoid undesirable reduction-oxidation reactions of chemical species in the electrolyte. Because of this, carrier drift velocity is typically well below the saturation velocity. Saturation velocity is included nonetheless for completeness.

### 2.3. Graphene EGFET Small-Signal Model

**Figure 3:**
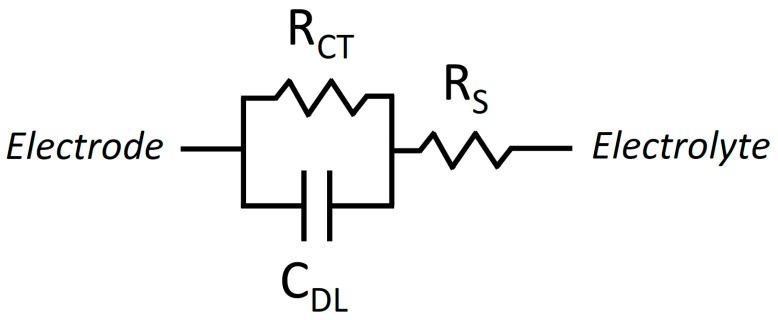
Schematic representation of the simplified Randles circuit commonly used to model electrode-electrolyte interfaces.

The graphene EGFETs under study are three terminal devices possessing source, drain, and gate terminals. All voltages and currents are referenced with respect to the source terminal making $V_{GS}$, $V_{DS}$, $I_{GS}$ and $I_{DS}$ an exhaustive list of the voltages and currents of interest. Small signal intrinsic voltage gain is defined as $A_{V}=g_{m}r_{o}=\partial V_{DS}/\partial V_{GS}$, where $g_{m}$ is the transconductance, which is defined as $\partial I_{DS}/\partial V_{GS}$, and $r_{o}$ is the output impedance defined as $\partial V_{DS}/\partial I_{DS}$. Development of a frequency-dependent graphene EGFET small-signal model requires an accurate model for electrode-electrolyte interfaces. This is accomplished using a simplified Randles circuit as given by Figure 3 [38,39].

**Figure 4:**
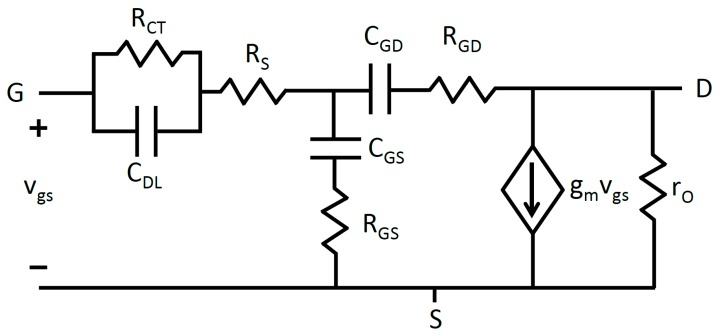
Graphene EGFET small-signal models depicting gate-source and drain-source capacitances and resistances using the simplified Randles circuit model.

$R_{CT}$ represents the charge transfer resistance, $R_{S}$ is the solution resistance, and $C_{DL}$ is the double layer capacitance. Electrode-electrolyte interfaces occur in three locations: the electrolyte-source interface, the electrolyte-drain interface, and at the reference electrode’s interface with the electrolyte. The simplified Randles circuit is substituted into the small-signal model at each of these locations. Graphene is known to possess a wide electrochemical window in electrolytic environments [11]. This translates into a very high charge transfer resistance $R_{CT}$, roughly on the order of GΩ. Because of this, $R_{CT}$ can be safely neglected at the gate-source and gate-drain terminals. This leads to the small-signal graphene EGFET model depicted in Figure 4.

**Figure 5:**
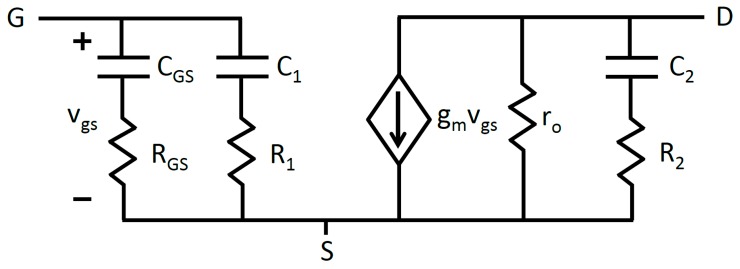
Final graphene EGFET small-signal model after application of the Miller theorem.

Electrochemistry experiments possess two interfaces, only one of which is the focus of study. In this case, the first interface exists between the reference electrode and electrolyte and the second interface occurs between the electrolyte and graphene EGFET. Reference electrodes are specifically designed to provide a stable reference potential and effectively translate changes in applied voltage entirely to the interface under study. This means that the reference electrode effectively translates the entirety of the small signal voltage $v_{gs}$ to the graphene EGFET and electrolyte interface. Because no series voltage drop occurs at the reference electrode, the simplified Randles circuit for the reference electrode may be neglected. Applying this fact in conjunction with the Miller theorem leads to the final small-signal model depicted in Figure 5.

This small-signal model for graphene EGFETs leads to the transfer function given by Equation (4).

(4) $$A_{v}\left(s\right)=-g_{m}r_{o}\frac{1+sR_{2}C_{2}}{1+s\left(R_{2}+r_{o}\right)C_{2}}$$

It becomes evident that the graphene EGFET small-signal model, unlike a Si-MOSFETs possesses a resistive component $R_{2}$ in series with the output parasitic capacitance. This unique component stems from the fact that graphene EGFETs are electrolyte-gated. Looking at the transfer function in the limits of low and high frequency operation produces the Equations (5) and (6), respectively.

(5) $$\underset{s\to 0}{lim}A_{v}\left(s\right)=-g_{m}r_{o}$$

(6) $$\underset{s\to \infty }{lim}A_{v}\left(s\right)=-g_{m}r_{o}\frac{R_{2}}{R_{2}+r_{o}}$$

$R_{1}$ and $C_{1}$ are given by Equations (7) and (8). Because gain $A_{v}$ is a negative value, the absolute value of the gain $\left|A_{v}\right|$ is used for clarity in showing how the magnitude of parasitic impedances are amplified at the input.

(7) $$R_{1}=R_{GD}\left(1+\left|A_{v}\right|\right)$$

(8) $$C_{1}=C_{GD}\left(1+\left|A_{v}\right|\right)$$

Similarly, equivalent impedances at the output are slightly reduced and given by Equations (9) and (10). This wholly details the development of the graphene EGFET small-signal model from first principles and provides the necessary reference equations describing individual model components.

(9) $$R_{2}=\frac{\left|A_{v}\right|}{1+\left|A_{v}\right|}R_{GD}$$

(10) $$C_{2}=\frac{\left(1+\left|A_{v}\right|\right)}{\left|A_{v}\right|}C_{GD}$$

## 3. Materials and Methods

### 3.1. Graphene EGFET Fabrication

**Figure 6:**
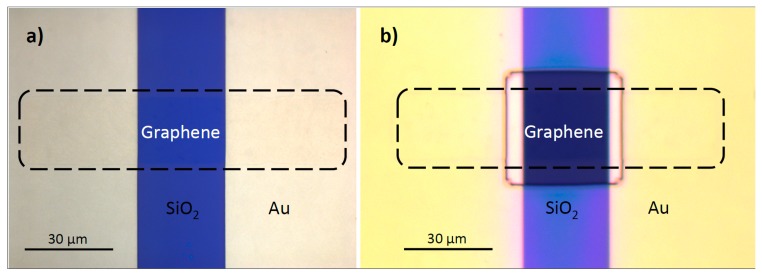
(**a**) Mesa etched graphene after removal of the bilayer MMA/SPR700 resist stack and (**b**) completely fabricated graphene EGFET with lead passivation using a recessed SU-8 layer.

Graphene EGFETs were fabricated on a piranha cleaned 4” thermally oxidized silicon wafer. Source and drain Ti/Au (10 nm/150 nm) contacts were patterned using lift-off photolithography. Monolayer graphene was then grown on copper foils using chemical vapor deposition (CVD) and transferred over the entire substrate [40]. The graphene channel regions were defined using MMA/SPR700 bilayer resist stacks and helium and oxygen plasma at 16 sccm and 8 sccm, respectively. Bilayer photoresist stacks were removed using acetone and isopropanol. The entire wafer was passivated with approximately 0.6 μm of SU-8 2000.5 and windows were photo defined to provide electrolyte access to the graphene EGFET channel regions. The SU-8 was hard-baked at 150 °C for five minutes to help remove cracks and pinholes. Figure 6 depicts a graphene EGFET at various stages in the fabrication process. An aqueous electrolyte droplet of 100 mM NaCl was pipetted over the graphene EGFET channel regions and a reference electrode was inserted into the droplet to gate the devices. Aqueous 100 mM NaCl was chosen because of its charge symmetry and similarity to physiological osmolarity.

### 3.2. Experimental Setup

Two experimental setups were employed for graphene EGFET characterization: one for DC characterization and one for AC characterization. DC characterization was performed to measure the graphene EGFET drain-source current $I_{DS}$ and a function of $V_{DS}$ and $V_{GS}$. This enables the calculation of transconductance, output impedance as well as intrinsic voltage gain. DC characterization also provides an independent means for measuring intrinsic voltage gain. In that way, DC and AC voltage gains can be compared and verified as consistent. AC characterization was performed by applying a small-signal voltage $V_{GS}$ to a common-source graphene EGFET amplifier and measuring the resulting output voltage $V_{DS}$ as a function of frequency.

The graphene EGFET experimental data is obtained from a device with dimensions W/L = 30 μm/30 μm and recessed passivation such that approximately 10 μm of the drain and source contacts were exposed to electrolyte. The device was measured in 100 mM aqueous NaCl electrolyte. DC measurements employed a platinum wire pseudo reference electrode for convenience. AC measurements require the use of a Ag/AgCl reference electrode. All measurements are taken at room temperature under ambient conditions with normal ventilation. The volume of the electrolyte droplet was monitored throughout the experiment and did not decrease appreciably indicating constant electrolyte concentration over the course of measurements.

## 4. Results

### 4.1. DC Characterization

**Figure 7:**
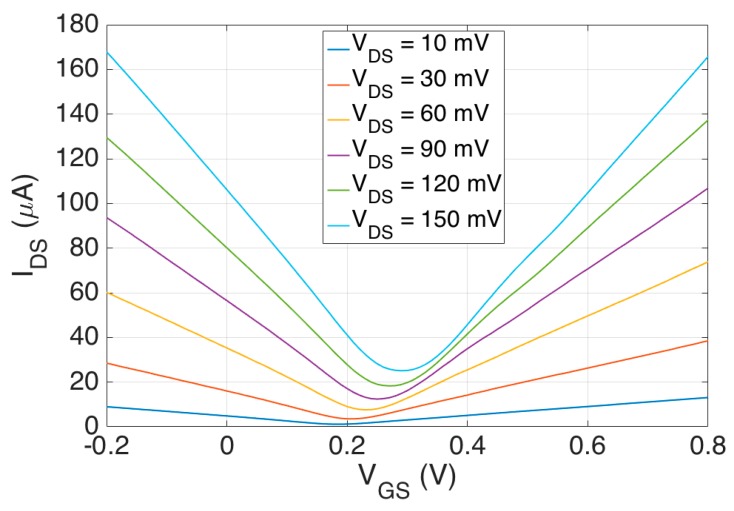
Graphene EGFET $I_{DS}$ vs. $V_{GS}$ for different applied $V_{DS}$ values.

DC data was acquired by sweeping $V_{GS}$ from −0.2 to 0.8 V and $V_{DS}$ from 10 mV to 150 mV. The step size was 10 mV for both $V_{GS}$ and $V_{DS}$. The $V_{GS}$ step rate was 500 ms per 10 mV. A ten second hold time was allotted when resetting $V_{GS}$ from 0.8 V to −0.2 V and incrementing $V_{DS}$ by 10 mV. Further increasing the hold time and decreasing the sweep rate had little effect on the DC curves meaning sufficient time was given for the ions to redistribute at the graphene-electrolyte interface and for the electric double layer to reach steady state. Full DC characterization consists of over 1500 data points. A conventional representation of the graphene EGFET DC characteristic is presented in Figure 7.

**Figure 8:**
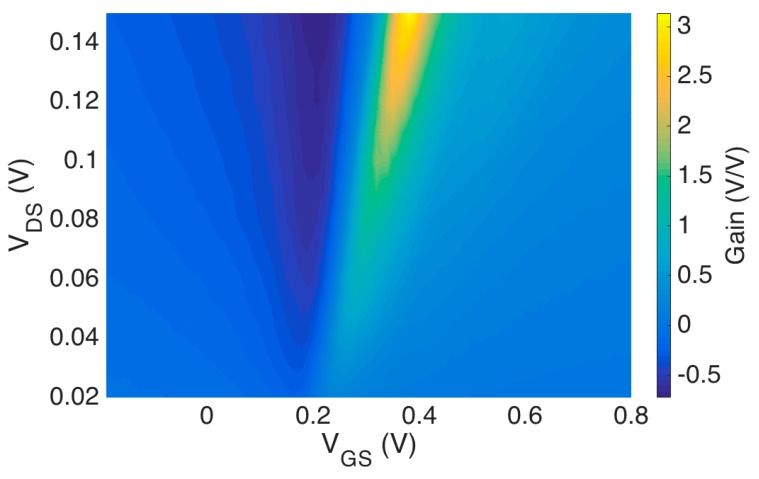
Intrinsic voltage gain as a function of $V_{DS}$ and $V_{GS}$ as calculated from DC characterization.

Intrinsic gain for the graphene EGFET was calculated by taking partial derivatives with respect to $V_{GS}$ and $V_{GS}$. Recall that intrinsic gain $A_{V}=g_{m}r_{o}=\partial V_{DS}/\partial V_{GS}$, where $g_{m}$ is $\partial I_{DS}/\partial V_{GS}$ and $r_{o}$ is $\partial V_{DS}/\partial I_{DS}$. Partial derivatives of the graphene DC characteristic are calculated numerically using finite differences to produce Figure 8.

### 4.2. Graphene EGFET Frequency Response

The frequency response of graphene EGFETs was investigated using the common-source (CS) amplifier configuration. In this way graphene EGFET frequency response is investigated while simultaneously demonstrating a graphene EGFET as functional amplifiers for the first time. A 98.99 kΩ resistor was employed as the drain resistor $R_{D}$. The operating voltage $V_{CC}$ was approximately 3.3 V and the drain of the graphene EGFET was biased at approximately 150 mV. Graphene EGFETs are not biased at high $V_{DS}$ voltages to avoid undesirable redox reactions at the graphene-electrolyte interface and potential damage to the graphene channel. A small-signal 20 mV p-p sinusoid $v_{in}$ was superimposed on a DC $V_{GS}$ bias. The DC bias was then manually adjusted to maximize the output $v_{o}$ and small-signal gain of the amplifier. The optimal $V_{GS}$ bias was found to be to the right of the Dirac point on the graphene I–V curve, which is consistent with Figure 8. This indicates that the graphene EGFET channel is n-type and that the transconductance is positive with respect to the orientation depicted in Figure 5. The frequency of the small-signal input voltage $v_{in}$ was then swept from 10 Hz to 50 kHz in order to characterize the CS amplifier’s frequency-dependent magnitude response. The CS amplifier transfer function closely resembles the transfer function of the intrinsic graphene EGFET derived in Equation (4). The key exception is that the CS amplifier contains an additional drain resistor $R_{D}$ at the output, which leads to the CS amplifier transfer function given by Equation (11).

(11) $$G_{v}\left(s\right)=-g_{m}\left(r_{o}//R_{D}\right)\frac{1+sR_{2}C_{2}}{1+s\left[R_{2}+\left(r_{o}//R_{D}\right)\right]C_{2}}$$

**Figure 9:**
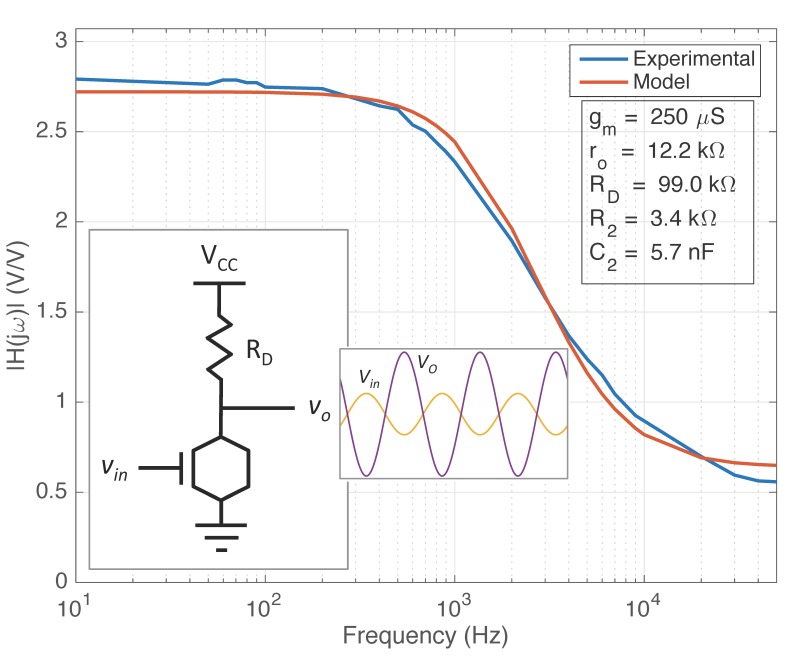
Fit of experimental graphene EGFET magnitude response with newly developed small-signal model for graphene EGFETs.

**Figure 10:**
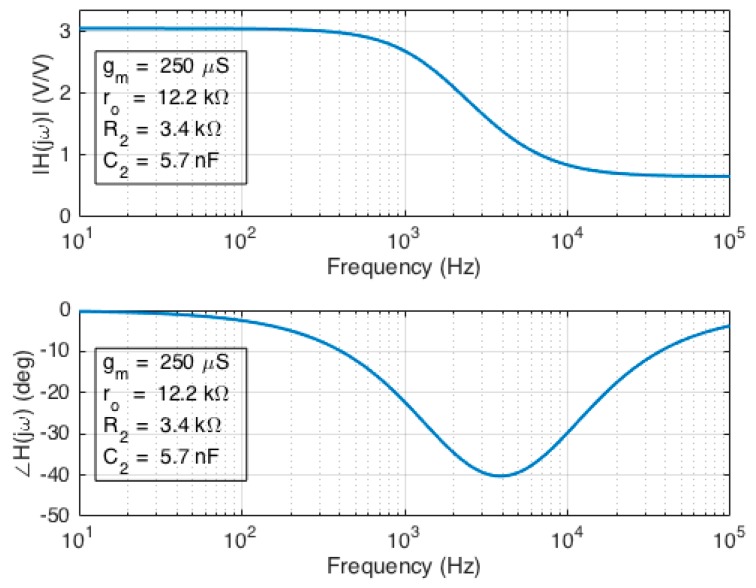
Bode plot depicting the intrinsic graphene EGFET magnitude and phase response.

The measured CS amplifier magnitude response was fit to the newly developed small-signal model for graphene EGFETs as shown by Figure 9. The CS amplifier reference schematic is provided as an inset in Figure 10 for convenience. Fitting was achieved using bounded simulating annealing in conjunction with a least squares error function. Transconductance was estimated at 250 μS from the DC characterization data previously obtained for a $V_{DS}$ operating bias of 150 mV. Small-signal model parameters, $r_{o}$, $R_{2}$, and $C_{2}$ were extracted as 12.2 kΩ, 3.4 kΩ, and 5.7 nF, respectively. The experimental data verifies the presence of parasitic capacitance $C_{2}$, which is responsible for the roll-off in gain. The experimental magnitude response also verifies the presence of resistance $R_{2}$ at the output, a unique feature in graphene EGFETs stemming from electrolyte solution resistance $R_{S}$. Maximum gain, cutoff frequency, and transition frequency were found to be approximately 2.8 V/V, 2.0 kHz, and 7.8 kHz, respectively.

The intrinsic magnitude response of the graphene EGFET is readily computed due to its similarity to the CS amplifier magnitude response (Equations (4) and (11)). Corresponding intrinsic phase response was computed using the extracted parameter values previously listed. A Bode plot for the intrinsic graphene EGFET magnitude and phase response is shown in Figure 10. The maximum intrinsic gain was found to be 3.1 V/V, the cutoff frequency was 1.9 kHz, and the transition frequency occurs at approximately 6.9 kHz. The corresponding equations for intrinsic graphene EGFET magnitude response and phase response are provided by Equations (12) and (13).

(12) $$||A_{v}\left(j\omega \right)||=g_{m}r_{o}\frac{\sqrt{1+{\left(\omega R_{2}C_{2}\right)}^{2}}}{\sqrt{1+{\left[\omega \left(R_{2}+r_{o}\right)C_{2}\right]}^{2}}}$$

(13) $$∠A_{v}\left(j\omega \right)=\frac{{tan}^{-1}\left(\omega R_{2}C_{2}\right)}{{tan}^{-1}\left[\omega \left(R_{2}+r_{o}\right)C_{2}\right]}$$

It is important to note that the CS amplifier small-signal equivalent circuit model neglects series resistance introduced by contact resistances. Previous work establishes the contact resistance at 6.3 kΩ μm for this graphene EGFET microfabrication process, which translates to a small additional series resistances of approximately 200 Ω per contact [35]. Gated contact resistances are accurately approximated as linear over a small voltage range, which is precisely the case for the small-signal $V_{GS}$ modulation of 20 mVp-p. Including contact resistance in the CS amplifier small-signal equivalent circuit model results in the DC gain described by Equation (14).

(14) $$\underset{s\to 0}{lim}G_{v}\left(s\right)=-\frac{g_{m}R_{D}}{1+g_{m}R_{C2}+\frac{R_{C1}}{r_{o}}+\frac{R_{C2}}{r_{o}}+\frac{R_{D}}{r_{o}}}$$

$R_{C1}$ and $R_{C2}$ represent the series resistances at the drain and source terminals, respectively. Because $g_{m}R_{C2}\ll 1$ and contact resistances $R_{C1}$ and $R_{C2}$ are much less than $r_{o}$, Equation (14) reduces to the DC gain previously described in Equation (11). Thus, contact resistances may be safely neglected in small-signal circuit models used to calculate amplifier gain and frequency response.

### 4.3. Performance Insights and Tradeoffs

Examination of parasitic capacitance $C_{2}$ provides further insight into the performance tradeoffs affecting graphene EGFET frequency response. Parasitic capacitance $C_{2}$ occurs between the drain and source. The extracted value of 5.7 nF for $C_{2}$ is too large to result from the graphene-electrolyte interface capacitance, which was previously approximated at 3 μF/cm^2^ as detailed in Section 2.1. Given the channel dimensions, the graphene-electrolyte interface contributes approximately 13.5 pF of parasitic gate-drain capacitance.

**Figure 11:**
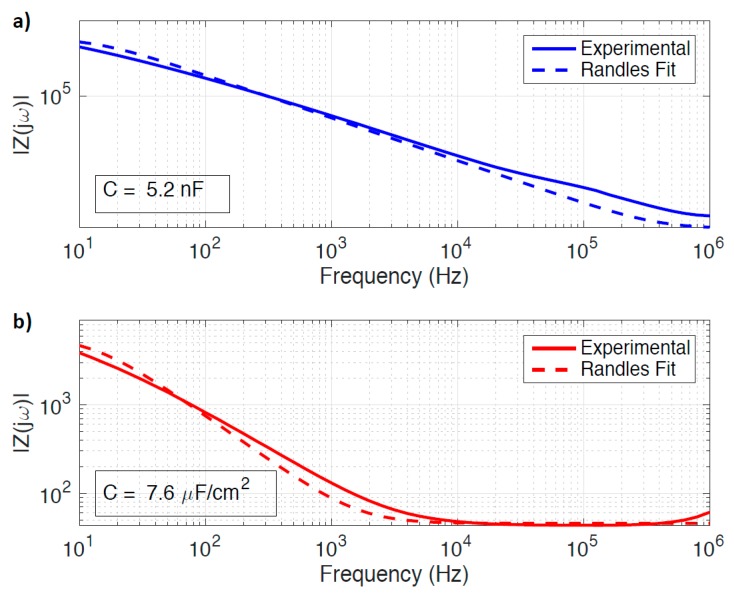
Electrochemical impedance spectroscopy (EIS) of (**a**) the drain contact lead with the graphene channel removed, and (**b**) the Au-electrolyte interface capacitance. Measurements were taken in aqueous 100 mM NaCl.

Figure 11A shows that the drain contact lead accounts for the majority of the parasitic capacitance $C_{2}$. Long contact leads are required to connect source and drain regions of the graphene EGFET, which are immersed in an electrolyte droplet, to dry contact pads that must be located further away (i.e., not submerged in electrolyte). A capacitance of 5.2 nF was extracted using EIS for the drain contact lead. This value is roughly equivalent (and consistent) with the 5.7 nF value of $C_{2}$ extracted using the newly developed small-signal model. Thus, contact lead capacitance greatly hampers graphene EGFET frequency response and motivates the development of smaller area leads.

Figure 11B shows the Au-electrolyte interface capacitance for the exposed metal of the drain region is approximately 7.6 μF/cm^2^. This is roughly 150% larger than the graphene-electrolyte interface capacitance and contributes approximately 40 nF in parasitic capacitance given the dimensions of the exposed drain metal. This results in an important tradeoff. Recessed channel passivation is known to reduce series resistance and enhance graphene EGFET transconductance performance. Recessed passivation, however, also introduces a parasitic capacitance substantially greater than that of the graphene-electrolyte interface. This motivates tighter misalignment constraints to reduce the metal exposure of the drain region. This finding also motivates development of self-aligned fabrication processes.

Lastly, attention should be drawn to the fact that $R_{2}$ introduces a pole in the graphene EGFET transfer function and ultimately controls the degree of gain degradation seen at high frequencies. $R_{2}$ stems from the solution resistance and therefore may be manipulated to a limited extent in many chemical and biological sensing applications. For instance, applications in which graphene EGFETs are employed as ion-selective chemical sensors (e.g., Na^+^, K^+^, Cl^−^, Ca^2+^) must necessarily vary ion concentrations and therefore solution resistance. Similarly, biological sensing applications provide little control over the cell medium composition and resulting solution resistance. This further motivates the reduction of parasitic capacitances, especially $C_{2}$ in order to enhance the frequency response of graphene EGFETs.

## 5. Conclusions

To the extent of our knowledge, this work develops the first small-signal frequency-dependent model for graphene electrolyte-gated field-effect transistors (EGFETs). This was accomplished by incorporating the Randles circuit into the small-signal field-effect transistor model. The newly developed small-signal model was shown to be capable of fitting the experimental data exceptionally well. Extracted parameters from the small-signal model were in good agreement with the parameters independently derived from DC characterization. Thus, two separate methods were employed to extract parameters, both of which yielded similar results. The small-signal model shows that graphene EGFETs contain a unique additional resistive element in series with the parasitic output capacitance. This added resistive element adds a zero to the transfer function causing the graphene EGFET magnitude response to level off at high frequencies. The presence of this additional pole was experimentally verified. All of these reasons attest to the accuracy of the newly developed small-signal model for graphene EGFETs.

This work also employs graphene EGFETs as a common-source amplifier configuration. To the extent of our knowledge, this work demonstrates for the first time, the ability of graphene EGFETs to function effectively as amplifiers providing a gain of 3 V/V. This concretely demonstrates the utility of graphene EGFET sensors in chemical and biological applications.

The majority of the parasitic drain-source capacitance $C_{2}$ was found to stem from the access lead for the drain. Contributions of the graphene-electrolyte interface and exposed metal drain contact are measured and compared as well. This reveals an important tradeoff in graphene EGFET design. Recessed channel passivation reduces parasitic series resistance and enhances graphene EGFET transconductance. However, recessed channel passivation also necessarily exposes some portion of the source and drain contacts. This increases parasitic capacitances and diminishes the operating frequencies for graphene EGFETs. This motivates the development of self-aligned microfabrication processes for high-performance graphene EGFETs.

This work provides a number of insights into the frequency-dependent small-signal behavior of graphene electrolyte-gated field-effect transistors (EGFETs). As such, it marks a critical step in the development of high-speed chemical and biological sensors based using graphene EGFETs.
